# Pandemic Critical Care Research during the COVID-19 (2020-2022): A Bibliometric Analysis Using VOSviewer

**DOI:** 10.1155/2022/8564649

**Published:** 2022-11-21

**Authors:** Mohammed Ageel

**Affiliations:** College of Medicine, Jazan University, Jazan 45142, Saudi Arabia

## Abstract

This paper has reviewed the global research on the pandemic critical care research during the COVID-19 from 2020 to 2022. To this end, a bibliometric and cluster analysis by full counting has been carried out using VOSviewer software and bibliographic data extracted from the Scopus database. The research found and studied 2778 documents. The types of research documents were limited to an article (81.46%), a letter (9.43%), an editorial (3.92%), a note (3.92%), a conference paper (0.90), and a short survey (0.04%). The results show an incessant increase in the number of research documents published and citations received during the COVID-19 pandemic. The U.S., U.K., Italy, and France have been shown to be the most productive countries, and there is a predominance of European institutions supporting and fostering research on pandemic critical care. Cecconi, M. (Italy) and Shankar-Hari, M. (U.K.) produced the highest number of research documents. Mapping of citation, co-citation, co-authorship, and keyword cooccurrence highlighted the hotspot, knowledge structure, and important themes. Citation dynamics for the top-cited research documents revealed static discourse. By reviewing the evolutionary trends of pandemic critical care research investigated factors, such as the influential works, main research topics, and the research frontiers, this paper reveals the scientific literature production's main research objectives and directions that could be addressed and explored in future studies. This paper reveals the scientific literature production's main research objectives and directions that could be addressed and explored in future studies after reviewing the evolutionary trends of pandemic critical care research during the COVID-19 and the investigated factors, such as influential works, main research topics, and research frontiers.

## 1. Introduction

According to the World Health Organization, a health system's three primary tasks are improving population health, providing financial protection against the expenses of illness, and reacting to people's expectations [1]. All three of these functions may benefit from critical care. In the United States, the majority of hospitals have one or more critical care units (ICU). Despite accounting for just 5% of hospital beds, ICUs in the United States use 15% to 20% of hospital budgets, equating to 1% of GDP [2]. Coronavirus disease 19 (COVID-19) has offered unprecedented problems to the healthcare system, some of which may result in significant change [3]. As healthcare personnel and policymakers recognize, a successful critical care surge response must be nested within the broader care delivery paradigm [4]. The pandemic of COVID-19 has brought to light important aspects of disaster planning. These include strategic reserves of personal protective equipment, intensive care unit (ICU) devices, consumables, and medications at the national or regional level and efficient supply chains and usage processes [5]. ICUs must also be prepared to handle spikes in patient numbers, and staffing models in ICUs should account for demand changes. Creating, implementing, and updating preexisting ICU triage and end-of-life care standards is necessary. Remote links with diverse healthcare staff and regular contact with family should be included in daily workflow operations. The COVID-19 epidemic reminds us that we must also strive to improve in addition to our responsibility to care [6].

Researchers and scientists do research to help critically sick patients avoid problems and improve their outcomes. They are always looking for novel diagnostic and therapy alternatives for persons suffering from severe diseases and trauma. Perioperative critical care, trauma, transfusions, modeling of critical care syndromes, life support therapies, patient-important outcomes, and health care delivery and informatics are areas of critical care research [7, 8].

The use of mathematical and statistical approaches to papers, books, and other forms of communication employed in the study of scientific publications is referred to as bibliometrics. Bibliometric approaches are often used to analyze scientific articles to discover research trends. Bibliometric techniques are a typical research tool for the systematic study of publications and have been used to quantify scientific progress in many areas of science and engineering [9, 10]. However, no bibliometric analysis of publications on pandemic critical care and COVID-19 has been published till now. As the COVID-19 pandemic has not been entirely under control and more knowledge should be obtained from these references, a bibliometric analysis is in critical need. Therefore, our study was performed timely to provide a broad understanding of pandemic critical care and future research directions.

## 2. Materials and Methods

The data for this paper were derived from the online version of the Scopus database. Bibliometric analyses are reflections of the coverage of their underlying databases in that the coverage basically specifies what is included in the study, and bibliometric assessment contextualizes these articles further against the database, which is likewise reliant on the coverage [11]. Scopus was established by Elsevier in 2004. Scopus, according to Elsevier, is the “most comprehensive overview of the world's research outputs,” overseen by a team of subject matter experts. Scopus aims to build the biggest feasible database of high-quality research articles. Scopus thus varies from WoS in that WoS prioritizes number above quality, while Scopus seeks a balance between the two. Elsevier and Clarivate Analytics both provide subscription-based databases [12, 13].

**Figure 1:**
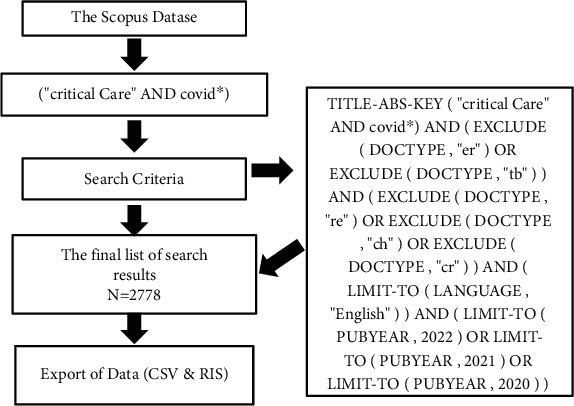
Search strategy in the Scopus database.

The titles of some of the published articles in the Scopus database were checked for a list of the utilized keywords to confirm that the bibliographic data was relevant to the study subject. [TITLE-ABS-KEY (“critical Care” and covid∗)] utilized a Wildcard (∗) and a Boolean operator (OR) with a combination of keywords [TITLE-ABS-KEY (“critical Care” and covid∗)].  Table 1 shows the preliminary findings, including articles, letters, notes, editorials, conference papers, and short surveys (2020-2022). As indicated in Figure 1, the findings were fine-tuned, and bibliometric data for all English-language research papers was acquired. Data for 2,778 articles (2020-2022) was downloaded into a Microsoft Excel spreadsheet in CSV format as the final result.

VOSviewer is a program that was used to create and visualize bibliometric networks. These networks comprise journals, researchers, or individual articles, and they were created based on citation, bibliographic coupling, co-citation, or co-authorship interactions [14]. Text mining capabilities are also included in VOSviewer, which was used to create and display cooccurrence networks of relevant phrases [15] retrieved from these 2,778 research documents. When creating a map using bibliographic data, the Create Map wizard in VOSviewer gives you the option of using one of two counting methods. Full counting is used in this study. Another option is to use fractional counting. GraphPad Prism software was utilized to display annual numbers of publications.

## 3. Results and Discussion

Coronavirus disease 19 (COVID-19) has presented the healthcare system with unprecedented challenges, some of which may result in considerable change [5]. Healthcare professionals and policymakers realize that an effective critical care surge response must be nested into the broader care delivery paradigm. The COVID-19 pandemic has brought to light critical elements of catastrophe preparation. The COVID-19 outbreak serves as a reminder that we must strive to improve in addition to our obligation to care. Researchers and scientists do studies to assist critically ill patients in avoiding complications and improving their prognosis [16]. *Bibliometric approaches* are a common research tool used to analyze publications and evaluate scientific progress systematically [9]. As a result, our study was conducted at the appropriate moment to give a comprehensive picture of pandemic critical care and future research prospects.

### 3.1. Overview of Pandemic Critical Care Research (2020-2022)

**Figure 2:**
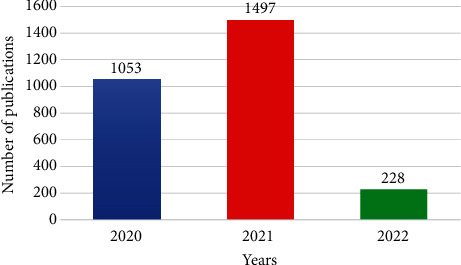
Temporal distribution of research documents.

Table 1 shows the preliminary findings from 2020 to 2022, including articles, letters, notes, editorials, conference papers, and short surveys. A total of 2,778 research documents in English were utilized for this bibliometric study (2020–2022). The distribution of research production (2020-2022) is presented in Figure 2, where the year 2021 represented 53.88% of the total research related to critical care during the Corona pandemic. The number of papers for 2022 (*N* = 228) until the middle of March. The types of research documents were limited to an article (81.46%), a letter (9.43%), an editorial (3.92%), a note (3.92%), a conference paper (0.90), and a short survey (0.04%). The low number of conference papers may result from imposed travel restrictions and social distancing that limited scientific gatherings. It expects to increase due to the presence of electronic platforms that provide all communication needs, including meetings, seminars, and online events.

**Figure 3:**
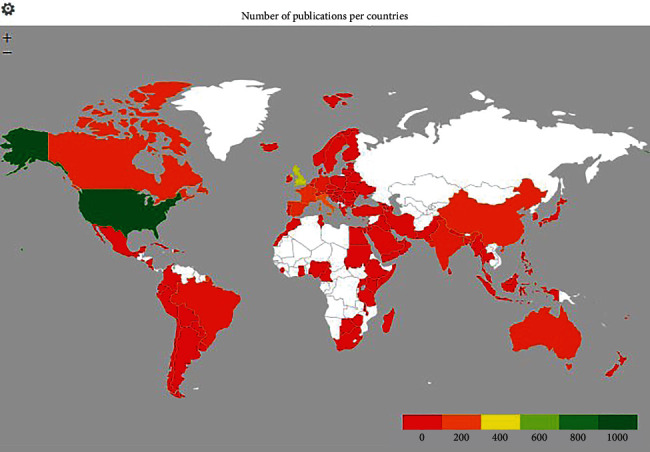
The geographic density based on the number research documents.

One hundred and twenty countries participated in the research related to pandemic critical care during COVID-19. The United States (31.5%), the United Kingdom (17.10%), Italy (8.24%), France (6.26%), Spain (5.62%), China (5.26%), Canada (5.22%), Australia (4.61%), India (4.39%), and Germany (4.21%) had more than a hundred scholarly discourses. The geographic density of research documents is shown in Figure 3. The top-productive organization is Harvard Medical School (*N* = 72), followed by Inserm (USA), University of Toronto (Canada), AP-HP Assistance Publique-de Paris (France), University of Oxford, Imperial College London, and King's College London (UK) with a threshold of more than fifty research documents. Cecconi, M. and Shankar-Hari, M. produced the highest number of research documents (*N* = 14). Cecconi is affiliated to the Department of Anesthesia and Intensive Care, Humanitas Clinical and Research Center-IRCCS, Italy. In 2020, Maurizio Cecconi was labeled one of three “pandemic heroes” by the Journal of the American Medical Association. As Italy confronted the first epidemic in the West, his early dedication to knowledge exchange and information dissemination was critical in slowing the virus's spread [17]. His research interest was on septic shock, hospital-acquired infections, the impact of frailty, use of Lopinavir-ritonavir and hydroxychloroquine, utilization of convalescent plasma, and noninvasive ventilatory support in critically ill patients with COVID-19 [18–21]. Shankar-Hari of King's College London spent a lot of time researching how to improve outcomes in adult critically sick patients with sepsis and ARDS by integrating disease immunobiology to interventional trial design [22, 23].

### 3.2. Collaborative Research and Co-Authorship Network

**Figure 4:**
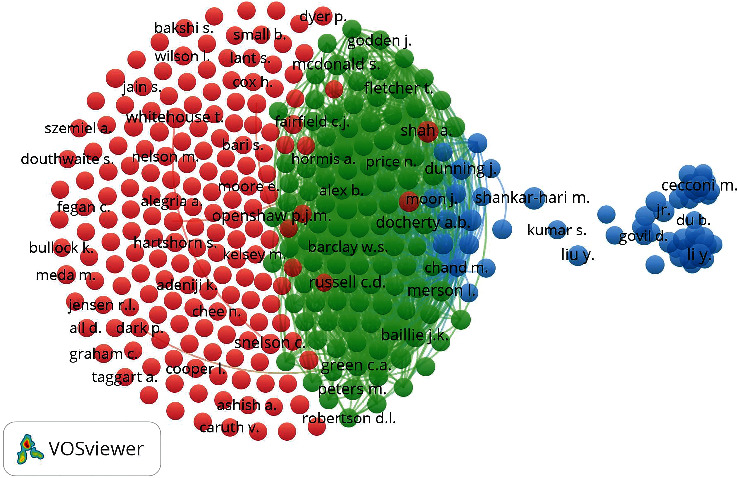
Network of main authors according to their co-authorship.

Collaborative scientific research is a critical component of contemporary academic landscapes spanning disciplines and research sectors and a significant research issue. Scientific cooperation may be described as an interaction between two or more scientists that occurs within a social environment and allows the exchange of meaning and accomplishing tasks related to a mutually shared, superordinate purpose. Collaboration may be seen as a technique for addressing the rising complexity and specialization of scientific research and the need for inter-and multidisciplinarity within these networks. Complex issue resolution often transcends typical disciplinary boundaries or necessitates an interdisciplinary approach. From another perspective, collaborative research enables increased productivity, as shown when writers cooperate with many co-authors or diverse research teams [24, 25]. Only under these circumstances is it feasible to maximize efficiency and production. The majority of contemporary research is the result of collaborative efforts, as indicated by the fact that the majority of articles are co-authored by two or more scholars [26]. The overall objective of this research was to evaluate the development and structure of scientific collaboration networks indicated by articles published on pandemic critical care between 2020 and 2022 and visualizes these networks visually. The collaborative research and co-authorship network were evaluated on three levels: authors, institutions, and countries. 29248 authors participated in pandemic critical care research during the COVID-19. The networks of the main scholars are shown in Figure 4. In certain networks, a central node exists that has a greater number of direct connections to other nodes. The benefit of this depiction of the major networks is that it enables a fast visualization of the major players and their relationships with one another. The significance of networks is determined by the size and number of their nodes and the thickness and number of the connections that connect them. 78 scholars were huddled using VOSviewer into three clusters with 46327 Links and a Total Link Strength of 367802. Authors from China assembled in blue the cluster. Shankar-Hari M. Shankar-Hari of King's College London is also placed in the blue cluster. The green cluster was colonized mostly by researchers from the ISARIC Global Support Centre at the University of Oxford, United Kingdom. ISARIC is the International Severe Acute Respiratory and Emerging Infection Consortium [27]. Scholars in the red clusters are mainly from ISARIC4C (Coronavirus Clinical Characterization Consortium), UK [28]. It could be said that the collaborative network on critical care research during the COVID-19 pandemic is mostly occupied by British and Chinese researchers.

**Figure 5:**
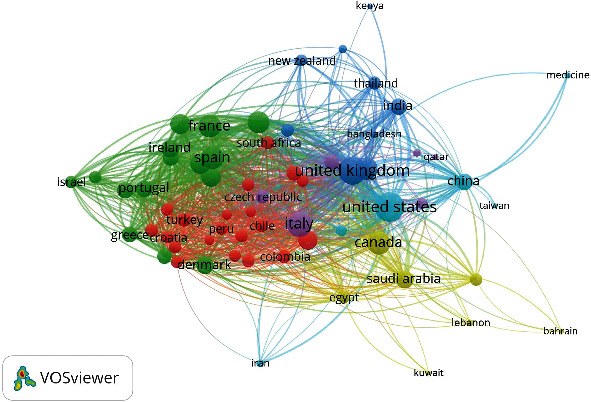
Network of main countries according to co-authorship.

The top-collaborative organization is Roslin Institute, University Of Edinburgh, Liverpool Clinical Trials Centre, University Of Liverpool, National Heart And Lung Institute, Imperial College London, Division Of Epidemiology And Public Health, University Of Nottingham School Of Medicine, Centre For Medical Informatics, Usher Institute, University Of Edinburgh, Centre For Tropical Medicine And Global Health, Nuffield Department Of Medicine, University Of Oxford, Intensive Care Unit, and Royal Infirmary Edinburgh (UK).  Figure 5 shows the collaborative network amongst countries (63) with a minimum number of five documents. US, UK, Italy, and China led 349 countries in their international collaboration in critical care research during the COVID-19 pandemic. 63 countries were clustered into six groups with 1292 Links and a Total Link Strength of 4480.

### 3.3. Research Impact: Citation Networking

**Figure 6:**
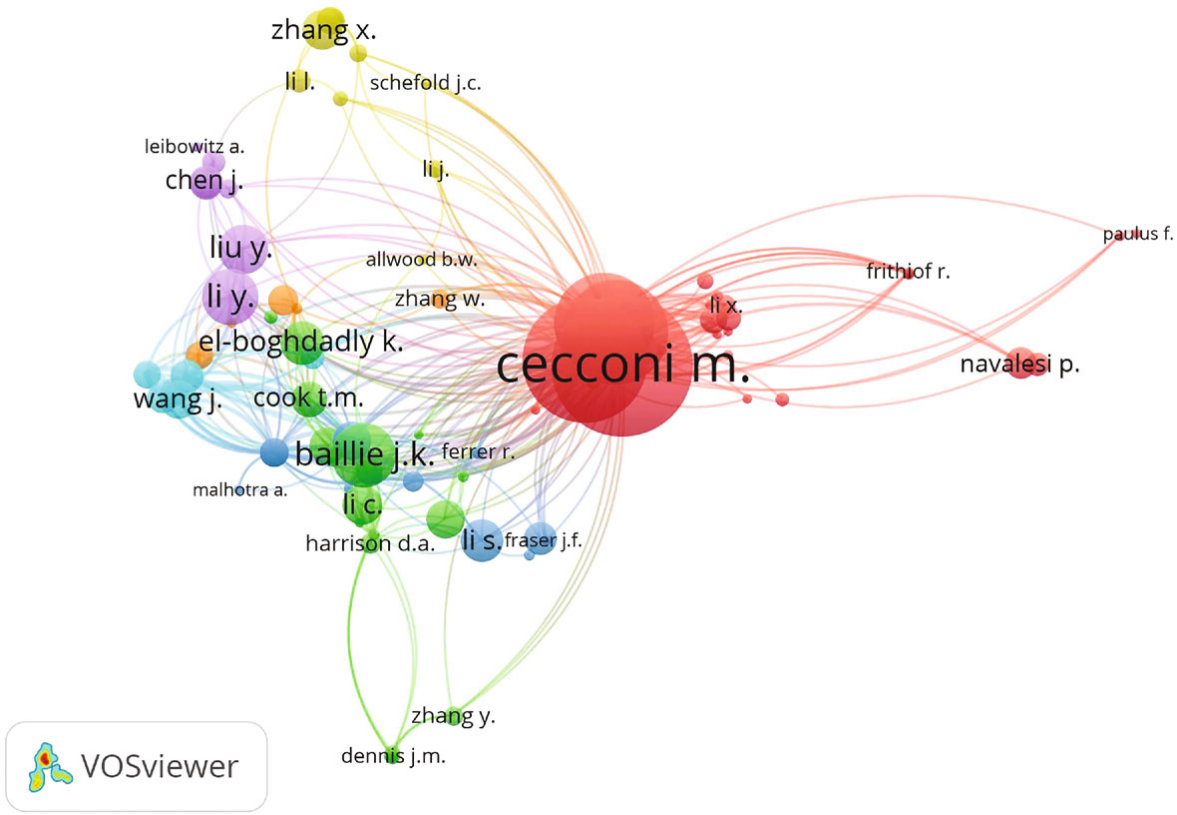
Citation network for authors.

Highly cited articles have a greater possibility of being seen, drawing the attention of other scholars. Evaluating the content of the most frequently referenced articles is quite beneficial for gaining insight into the trends in certain sectors in terms of research development. It may show scholars how to choose the right area or journal for their publication. Although citations are not a scientific technique for evaluating publications, they are an essential indicator for recognizing research parameters. The citation index, a form of bibliometric approach, indicates how often an article has been cited in other publications. Thus, citation analysis assists scholars in gaining a basic understanding of the articles and research that influence a certain area of study, and it is concerned with the investigation of the documents mentioned in academic works [29, 30].  Figure 6 and Table 2 show the most cited authors. Italy and the United Kingdom have the largest share in terms of the number of scientists' citations. The top four Italian authors work on noninvasive respiratory assistance outside the critical care unit for Coronavirus-19 disease-related acute respiratory failure [31]. Abbasi et al. [32] shows that researchers related to many diverse academics obtain more citations than researchers with fewer connections. As a result, it is critical to collaborate in successful research networks in performance.

**Figure 7:**
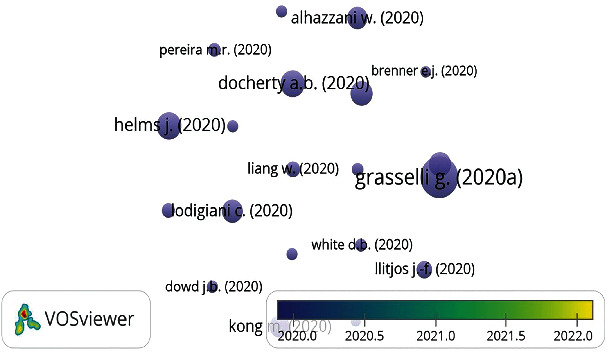
Citation dynamics for twenty research documents.

**Figure 8:**
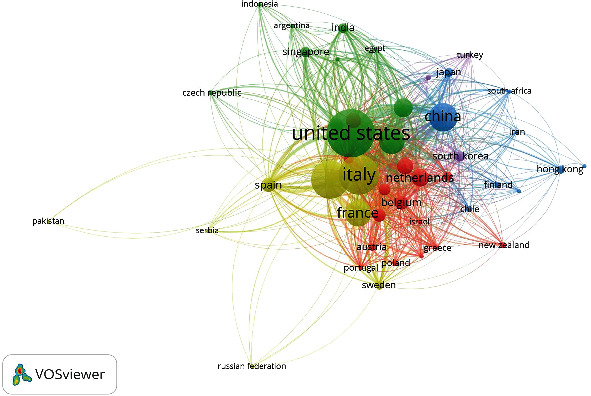
Citation network for countries.

Seven clusters were built using bibliographic data for 86 authors with a minimum citation of eight. Italian authors anchor the red cluster. The green cluster is led by Baillie J.K. of MRC Human Genetics Unit, Institute of Genetics and Cancer, University of Edinburgh, UK. Baillie's interest is in translational genomics in critical care medicine [33, 34]. Li J., Wang J., Zhang X., Li S. are the top-cited authors in the other clusters.  Table 3 presents the twenty most-cited research documents. Eleven of them were directly on the subject of this bibliometric paper. The top-cited article was on the Italian experience in critical care during the COVID-19 outbreak and the prediction during emergency response. This paper was published in the Journal of the American Medical Association, which included four papers from the total papers in Table 4. The citation dynamics for research documents were elicited using overlay visualization.  Figure 7 shows that all the top-cited articles began to have their knowledge impact since the year 2020, and it is noted that the nodes were not covered in yellow but instead maintained their purple color. Table 4 represents the top-impactful journals used in knowledge dissemination of pandemic critical care along with their number of documents, number of citations, WOS's impact factor, and Sopus's Citescore. The top-cited one is the Journal of The American Medical Association, with a total citation of 4632. IRCCS San Raffaele Scientific Institute, University of Milan (Italy), University of Edinburgh, and University of Liverpool (UK) are the top-cited organizations.  Figure 8 depicts the geographic distribution of impactful research on pandemic critical care. Countries based on citation were grouped into five clusters, but their grouping does not follow the geographical pattern. The Netherlands anchored the red cluster; while the United States led the group in green. China, South Korea, the United Kingdom, and Italy dominated the other clusters.

### 3.4. Co-Citation Analysis

**Figure 9:**
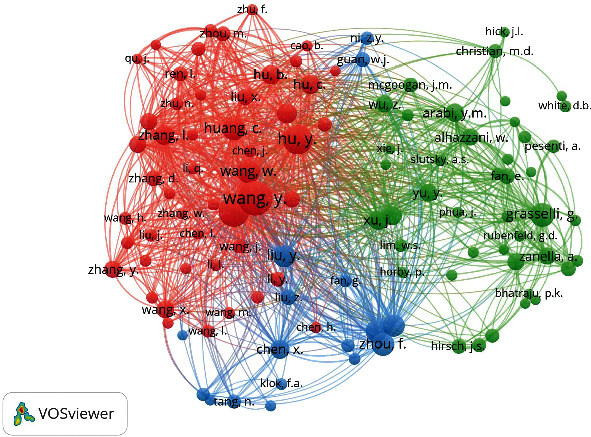
Visualization network of the of pandemic critical care research; co-citation analysis.

In order to undertake a complete and systematic study of pandemic critical care research, we also concentrated on bibliometric co-citation analysis. Co-citation analysis connects journals, authors, and other documents using citation methods to determine the origins of the field and its overall structure. The research is based on previously mentioned works to determine where a certain field began [42, 43]. To solve the following study topic, we performed co-citation analysis to identify the field's core, peripheral, and bridging researchers, and how has the organization evolved over time. Co-citation analysis of the 110 most co-cited authors (visualized in Figure 2) from 2020 to 2022 revealed three clusters. The first cluster (represented in green in Figure 9) consisted of 43 articles. Based on citations, links, and total link strength, the most influential authors in Cluster 1 were Grasselli, G., Arabi, Y.M., Alhazzani, W., Zanella, A., and Mcgoogan, J.M. Researchers in this cluster worked on the guidelines, response, utilization, resources, operationalization, and baseline parameters in pandemic critical care [44–47]. Wang, Y. Li, X., Hu, Y., Zhang, L., and Huang, C. are the to-cited authors in the red cluster. The authors identified the clinical characteristics and outcomes of critically ill patients with COVID-19 [48, 49]. Authors in the blue cluster worked on novel diagnostic and therapy alternatives for COVID-19 patients admitted to ICU [50]. It was noted that the research areas were not defined by clear knowledge boundaries and an intertwining between the three clusters was observed. This is explained by the fact that critical care during the pandemic received research and joint citation of an estimated number, which were insufficient to frame the well-defined research areas due to the short period. Nonpandemic critical care researchers are constantly on the lookout for better diagnostic and therapeutic approaches for patients suffering from severe diseases and trauma. Nonpandemic critical care research fields include perioperative critical care, trauma, transfusions, modeling of critical care syndromes, life support therapies, patient-important outcomes, and health care delivery and informatics [8].

### 3.5. Lexical Analysis: Keywords' Cooccurrence

**Figure 10:**
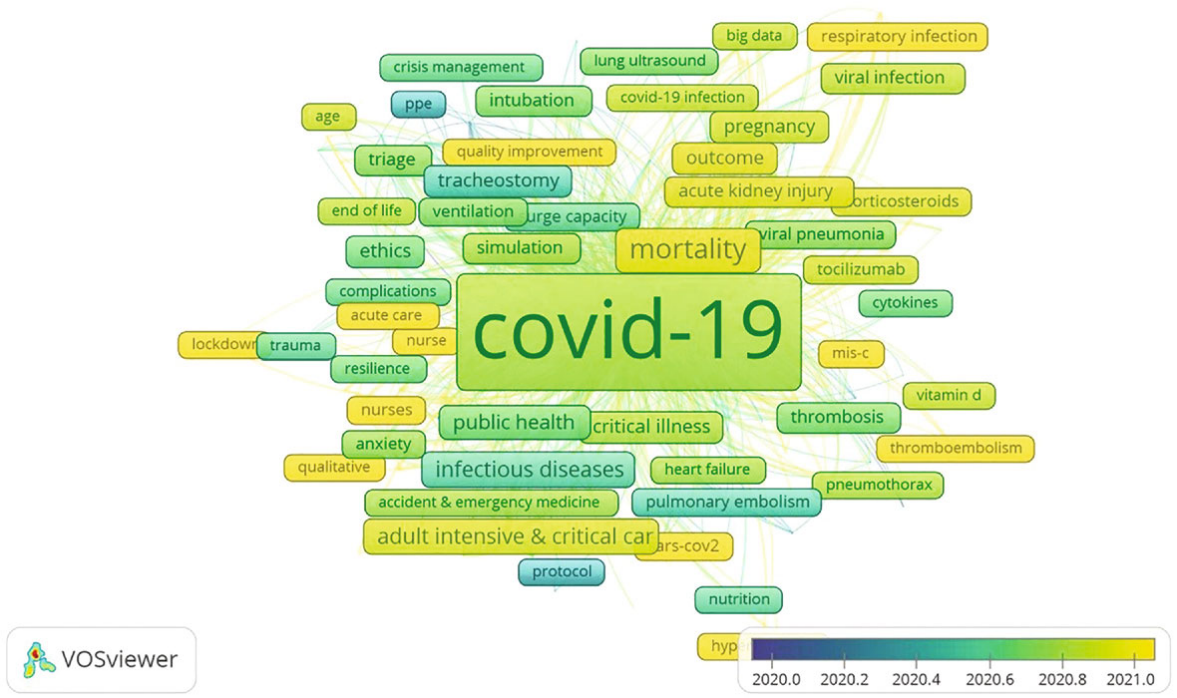
Overlay visualization of keywords cooccurrence and their survival patterns during 2020 and 2021. 2022 was not included in the analysis.

**Figure 11:**
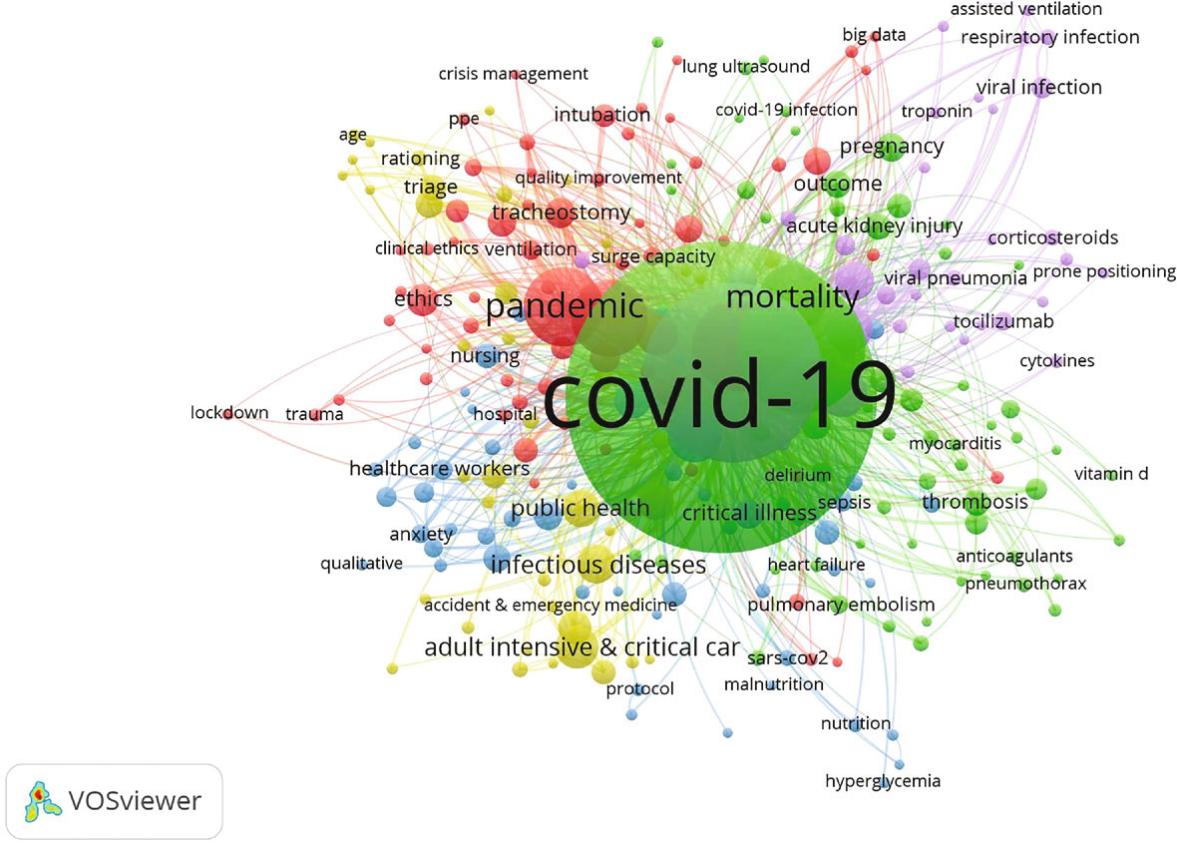
Cooccurrences of keyword network (min 5).

The following analysis was a coword analysis, with a word as the unit of analysis. Coword analysis is a bibliometric technique that identifies relationships between concepts that appear in the same title, abstract, or keyword. The advantage of this strategy is that it can generate a field and structure from the coword. However, the drawback is that words can take on a variety of forms and interpretations. The core premise of coword analysis is that words that frequently occur in documents may imply related concepts. Among the bibliometric methods, coword analysis is the only one that generates similarity metrics based on the actual text. Coword analysis scans keywords, titles, and abstracts to produce a semantic map of the field and demonstrate the key constructions around which the field is formed, historically and currently. Coword analysis has developed into a strategically important method for knowledge discovery in databases [51, 52]. We used the same database for the coword analysis that we used for the co-citation study. Only 265 of the 3785 keywords met our criterion of appearing at least five times.  Figure 10 shows the overlay visualization of keywords cooccurrences and their survival patterns during 2020 and 2021. 2022 was not included in the analysis. According to the color, scale, and frames Figure 11 with yellow color are emerging keywords. Five groupings emerged from the investigation. “COVID-19”, “critical care”, “pandemic”, “intensive care”, and “mortality” were the strongest phrases in terms of both number of linkages and total link strength. There were a total of 2,949 links and a total link strength of 8299 in all five clusters.

In Cluster 1 (green), the two most dominant words were COVID-19 and mortality. Cluster 2 (red) dealt with pandemic intensive care. Cluster 3 focused on mechanical ventilation, ARDS, pneumonia, and respiratory failure, which emerged as highly visible keywords. Public health and epidemiology anchored the keywords in cluster four (yellow). Cluster five was about mental health, nursing, rehabilitation, and stress.

Narrowing in on the specific topics identified by the coword analysis, besides the previously mentioned clusters, inter and intracluster were recognized as follows:

- Tracheostomy, extracorporeal membrane oxygenation, intubation, prone position, and airway management
- Resource allocation, surge capacity, ethics, health policy, education, length of stay, and outcomes
- Anxiety, patient safety, healthcare workers, qualitative research, burnout, personal protective equipment
- Case report, comorbidity, pregnancy, thrombosis, prognosis, acute kidney injury, inflammation, risk factors, sepsis, children, and venous thromboembolism
- Tocilizumab and corticosteroids
- Computed tomography and biomarkers
- Simulation, artificial intelligence, telemedicine, and machine learning

## 4. Conclusion

The global Coronavirus disease 2019 (COVID-19) pandemic has resulted in an influx of acute critical illness patients requiring basic and advanced life support in the intensive care unit [53]. To expand critical care capacity and maximize safety for everybody, critical care leaders have battled with difficult decisions about which services should be emphasized, which should be curtailed, and which should be stopped in preparation for the expected surge of patients with COVID-19 [54]. Although critical care research is always vital, it is a top priority worldwide during the COVID-19 pandemic. Quickly assembled or existing research teams, a responsive financing mechanism, speedy ethical and contract approval, and the commitment of research and bedside staff are all required to prioritize pandemic-specific research effectively [55]. To increase our understanding of pathophysiology, immunology, diagnosis, prognosis, prevention, treatment, triage, and palliation, observational research and randomized trials are required. The International Severe Acute Respiratory and Emerging Infection Consortium (ISARIC) was founded in 2012 with the goal of removing roadblocks to the study of emerging infectious illnesses by developing procedures and case report forms that could be quickly modified to new outbreaks [27]. Bibliometric techniques are a typical type of research tool used to assess scientific progress by systematically analyzing publications [56]. As a result, we conducted our research at the right time to provide a comprehensive picture of pandemic critical care and future research opportunities. VOSviewer is a program that was used to create and visualize bibliometric networks [57]. These networks comprise journals, researchers, or individual articles, and they were created based on citation, bibliographic coupling, co-citation, or co-authorship interactions. Text mining capabilities are also included in VOSviewer, which was used to create and display cooccurrence networks of relevant phrases retrieved from these 2,778 research documents. The top-collaborative organization is the University of Edinburgh, UK. Analysis of co-citation analysis and keywords cooccurrence revealed versatile knowledge structures. Keywords cooccurrence was analyzed (*Lexical Analysis*). And the data analysis revealed five distinct clusters. In terms of both the number of links and the overall link strength, the words “COVID-19,” “critical care,” “pandemic,” “intensive care,” and “mortality” were the strongest. There have never been such drastic threats to the healthcare system as COVID-19. Healthcare professionals and researchers alike recognize the need of situating critical care surge responses within broader systems of care. In view of the widespread spread of COVID-19, it is essential to have certain emergency plans in place. The pandemic has underlined the need of preexisting epidemiological registries and agile randomized controlled platform trials in the generation of rapid, trustworthy data. The COVID-19 epidemic is a reminder that in addition to our obligation to care, we are also dedicated to advancing. By addressing these issues now, we will be able to deliver superior treatment to future patients.

## 5. Limitations of the Study

This was the first attempt to map the pandemic critical care studies during the COVID-19 epidemic. However, there are certain limits to our bibliometric analysis to consider. For example, we only used one database (Scopus) and assessed impact based on Elsevier-covered citations. As a result, this procedure was constrained by the indexing requirements imposed by these databases and did not include all scientific journals [58]. Because pandemic critical care research during the COVID-19 is still in its early stages, it will take time for studies to be recognized and cited. We included peer-reviewed papers published in English to ensure sample homogeneity. Other publications in different languages would be useful in the future. Given that this is a young and rising topic, we established a minimal threshold level and built clusters based on that threshold; this is a limitation of this bibliometric research because some potentially intriguing publications may have been eliminated.

## Figures and Tables

**Table 1:** Preliminary research.

Document type	2020-2022	
	Number	Percent
Article	2263	81.46
Letter	262	9.43
Editorial	109	3.92
Note	109	3.92
Conference paper	25	0.90
Short survey	1	0.04
Total	2778	100

**Table 2:** Top cited authors in pandemic critical care research.

Rank	Author	Affiliation	N	C	C/N	TLS
1^st^	Cecconi M.	Department of Anaesthesia and Intensive Care Medicine, Humanitas Clinical and Research Centre-IRCCS, Italy	14	5802	414.43	193
2^nd^	Grasselli G.	Department of Pathophysiology and Transplantation, University of Milan, Italy	11	3887	353.36	247
3^rd^	Foti G.	General Intensive Care Unit, Emergency Department-ASST Monza-San Gerardo Hospital, University of Milano-Bicocca, Italy	10	3148	314.80	119
4^th^	Zangrillo A.	Department of Anaesthesia and Intensive Care Medicine, Humanitas Clinical and Research Centre-IRCCS, Italy	10	2850	285.00	109
5^th^	Dunning J.	National Heart and Lung Institute, Faculty of Medicine, Imperial College London, London, UK.	13	2010	154.62	209
6^th^	Baillie J.K.	MRC Human Genetics Unit, Institute of Genetics and Cancer, University of Edinburgh, UK	15	1952	130.13	222
7^th^	Harrison E.M.	Centre for Medical Informatics, Usher Institute, University of Edinburgh, UK.	14	1950	139.29	209
8^th^	Docherty A.B.	Centre for Medical Informatics, The Usher Institute, University of Edinburgh, UK.	14	1938	138.43	209
9^th^	Carson G.	Nuffield Department of Clinical Medicine, ISARIC Global Support Centre, Centre for Tropical Medicine and Global Health, University of Oxford, Oxford, UK.	11	1935	175.91	196
10^th^	Halpin S.	Academic Department of Rehabilitation Medicine, Leeds Institute of Rheumatic and Musculoskeletal Medicine, School of Medicine, University of Leeds, UK.	10	1935	193.50	182

N: number of publications; C: citations; C/N: citation average; TLS: total link strength.

**Table 3:** Top-cited documents in pandemic critical care research.

Rank	Article	Title	Journal	Citation	REF
1^st^	Grasselli G. [35]	Critical care utilization for the COVID-19 outbreak in Lombardy, Italy: early experience and forecast during an emergency response.	Journal of the American Medical Association	2596	[35]
2^nd^	Helms J. [36]	High risk of thrombosis in patients with severe SARS-CoV-2 infection: a multicenter prospective cohort study	Intensive Care Medicine	1305	[36]
3^rd^	Docherty A.B. [37]	Features of 20,133 UK patients in hospital with COVID-19 using the ISARIC WHO clinical characterisation protocol: prospective observational cohort study	The BMJ	1263	[37]
4^th^	Grasselli G. [38]	Baseline characteristics and outcomes of 1591 patients infected with SARS-CoV-2 admitted to ICUs of the Lombardy region, Italy	JAMA - Journal of the American Medical Association	1082	[38]
5^th^	Petrilli C.M. [39]	Factors associated with hospital admission and critical illness among 5279 people with Coronavirus disease 2019 in new York City: prospective cohort study	The BMJ	1068	[39]
6^th^	Lodigiani C. [40]	Venous and arterial thromboembolic complications in COVID-19 patients admitted to an academic hospital in Milan, Italy.	Thrombosis Research	1001	[40]
7^th^	Kong M. [41]	Multisystem inflammatory syndrome in U.S. children and adolescents	New England Journal of Medicine	935	[41]
8^th^	Alhazzani W. (2020)	Surviving Sepsis Campaign: guidelines on the management of critically ill adults with Coronavirus Disease 2019 (COVID-19)	Intensive Care Medicine	918	
9^th^	Llitjos J.-F. (2020)	High incidence of venous thromboembolic events in anticoagulated severe COVID-19 patients	Journal of Thrombosis and Haemostasis	601	
10^th^	Liang W. (2020)	Development and validation of a clinical risk score to predict the occurrence of critical illness in hospitalized patients with COVID-19	JAMA Internal Medicine	537	
11^th^	Cook T.M. (2020)	Consensus guidelines for managing the airway in patients with COVID-19: guidelines from the difficult airway society, the Association of Anaesthetists the Intensive Care Society, the Faculty of Intensive Care Medicine and the Royal College of Anaesthetists	Anaesthesia	454	
12^th^	Pereira M.R. (2020)	COVID-19 in solid organ transplant recipients: initial report from the US epicenter	American Journal of Transplantation	419	
13^th^	Cai Q. (2020)	COVID-19: abnormal liver function tests	Journal of Hepatology	390	
14^th^	White D.B. (2020)	A framework for rationing ventilators and critical care beds during the COVID-19 pandemic	JAMA - Journal of the American Medical Association	380	
15^th^	Spiezia L. (2020)	COVID-19-related severe hypercoagulability in patients admitted to intensive care unit for acute respiratory failure	Thrombosis and Haemostasis	365	
16^th^	Lippi G. (2020)	Cardiac troponin I in patients with coronavirus disease 2019 (COVID-19): evidence from a meta-analysis	Progress in Cardiovascular Diseases	365	
17^th^	Dowd J.B. (2020)	Demographic science aids in understanding the spread and fatality rates of COVID-19	Proceedings of the National Academy of Sciences of the United States of America	362	
18^th^	Murthy S. (2020)	Care for Critically ill Patients with COVID-19.	JAMA - Journal of the American Medical Association	361	
19^th^	Spyropoulos A.C. (2020)	Scientific and standardization committee communication: clinical guidance on the diagnosis, prevention, and treatment of venous thromboembolism in hospitalized patients with COVID-19	Journal of Thrombosis and Haemostasis	334	
20^th^	Brenner E.J. (2020)	Corticosteroids, but not TNF antagonists, are associated with adverse COVID-19 outcomes in patients with inflammatory bowel diseases: results from an international registry	Gastroenterology	330	

**Table 4:** Top-cited journals in pandemic critical care research.

Rank	Journal	N	C	IF	Citescore
1^st^	JAMA - journal of the American Medical Association	9	4632	56.30	24.8
2^nd^	Intensive care medicine	24	3095	17.44	4.5
3^rd^	The BMJ	8	2750	39.890	4.0
4^th^	Thrombosis research	9	1250	3.944	6.6
5^th^	Journal of thrombosis and hemostasis	6	1180	5.824	11.3
6^th^	New England journal of medicine	5	1094	91.245	80.6
7^th^	Anesthesia	22	1032	6.955	10.1
8^th^	Critical care	57	994	9.372	10.1
9^th^	Lancet respiratory medicine	17	882	30.700	43.7
10^th^	American journal of transplantation	7	775	8.086	11.4

N: number of publications; C: citations; IF: impact factor.

## Data Availability

Data is available upon request.

## References

[1] WHO (2000). The world health report 2000: health systems: improving performance.

[2] Luce J. M., Rubenfeld G. D. (2002). Can health care costs be reduced by limiting intensive care at the end of life?. *American Journal of Respiratory and Critical Care Medicine*.

[3] Druss B. G. (2020). Addressing the COVID-19 pandemic in populations with serious mental illness. *JAMA Psychiatry*.

[4] Rainey S., Mormina M., Lignou S., Nguyen J., Larsson P. (2021). The post-normal challenges of COVID-19: constructing effective and legitimate responses. *Science and Public Policy*.

[5] Ippolito M., Ramanan M., Bellina D. (2021). Personal protective equipment use by healthcare workers in intensive care unit during the early phase of COVID-19 pandemic in Italy: a secondary analysis of the PPE-SAFE survey. *Therapeutic Advances in Infectious Disease*.

[6] Anka A. U., Tahir M. I., Abubakar S. D. (2021). Coronavirus disease 2019 (COVID-19): an overview of the immunopathology, serological diagnosis and management. *Scandinavian Journal of Immunology*.

[7] Pronovost P. J., Angus D. C., Dorman T., Robinson K. A., Dremsizov T. T., Young T. L. (2002). Physician staffing patterns and clinical outcomes in critically ill patients: a systematic review. *JAMA*.

[8] Granholm A., Kaas-Hansen B. S., Kjær M. B. N. (2021). Patient-important outcomes other than mortality in recent ICU trials: protocol for a scoping review. *Acta Anaesthesiologica Scandinavica*.

[9] Lawani S. M. (1981). Bibliometrics: its theoretical foundations, methods and applications. *Libri*.

[10] Mavragani A., Ochoa G., Tsagarakis K. P. (2018). Assessing the methods, tools, and statistical approaches in google trends research: systematic review. *Journal of Medical Internet Research*.

[11] Borgman C. L., Furner J. (2002). Scholarly communication and bibliometrics. *Annual Review of Information Science and Technology*.

[12] Aghaei Chadegani A., Salehi H., Yunus M. (2013). A comparison between two main academic literature collections: web of science and Scopus databases. *Asian Social Science*.

[13] Norris M., Oppenheim C. (2007). Comparing alternatives to the *web of science* for coverage of the social sciences ' literature. *Journal of Informetrics*.

[14] Van Eck N., Waltman L. (2010). Software survey: VOSviewer, a computer program for bibliometric mapping. *Scientometrics*.

[15] Ding X., Yang Z. (2022). Knowledge mapping of platform research: a visual analysis using VOSviewer and CiteSpace. *Electronic Commerce Research*.

[16] Madara J., Miyamoto S., Farley J. E. (2021). Clinicians and professional societies COVID-19 impact assessment: lessons learned and compelling needs. *NAM Perspectives*.

[17] Paterlini M. (2021). Covid-19: Maurizio Cecconi—one year since Italy’s darkest moment. *BMJ*.

[18] Writing Committee for the REMAP-CAP Investigators (2021). Effect of convalescent plasma on organ support-free days in critically ill patients with COVID-19: a randomized clinical trial. *JAMA*.

[19] Evans L., Rhodes A., Alhazzani W. (2021). Surviving sepsis campaign: international guidelines for management of sepsis and septic shock 2021. *Critical Care Medicine*.

[20] Grasselli G., Scaravilli V., Mangioni D. (2021). Hospital-acquired infections in critically ill patients with COVID-19. *Chest*.

[21] Jung C., Flaatten H., Fjølner J. (2021). The impact of frailty on survival in elderly intensive care patients with COVID-19: the COVIP study. *Critical Care*.

[22] van der Poll T., Shankar-Hari M., Wiersinga W. J. (2021). The immunology of sepsis. *Immunity*.

[23] The WHO Rapid Evidence Appraisal for COVID-19 Therapies (REACT) Working Group (2021). Association between administration of IL-6 antagonists and mortality among patients hospitalized for COVID-19: a meta-analysis. *JAMA*.

[24] Heinze T., Kuhlmann S. (2008). Across institutional boundaries?: research collaboration in German public sector nanoscience. *Research Policy*.

[25] Razzaq S., Malik A. K., Raza B., Khattak H. A., Moscoso Zegarra G. W., Zelada Y. D. (2018). Research collaboration influence analysis using dynamic co-authorship and citation networks. *International Journal of Interactive Multimedia and Artificial Intelligence*.

[26] Gbey E., Turkson R. F., Lee S. (2022). A bibliometric survey of research output on wireless charging for electric vehicles. *World Electric Vehicle Journal*.

[27] ISARIC https://isaric.org/.

[28] ISARIC4 https://isaric4c.net/.

[29] Liu P.-C., Lu Y., Lin H.-H. (2022). Classification and citation analysis of the 100 top-cited articles on adult spinal deformity since 2011: a bibliometric analysis. *Journal of the Chinese Medical Association*.

[30] Shahid I., Motiani V., Siddiqi T. J. (2020). Characteristics of highly cited articles in heart failure: a bibliometric analysis. *Future Cardiology*.

[31] Cammarota G., Esposito T., Azzolina D. (2021). Noninvasive respiratory support outside the intensive care unit for acute respiratory failure related to coronavirus-19 disease: a systematic review and meta-analysis. *Critical Care*.

[32] Abbasi A., Hossain L., Uddin S., Rasmussen K. J. (2011). Evolutionary dynamics of scientific collaboration networks: multi-levels and cross-time analysis. *Scientometrics*.

[33] Dorward D. A., Russell C. D., Um I. H. (2021). Tissue-specific immunopathology in fatal COVID-19. *American Journal of Respiratory and Critical Care Medicine*.

[34] Maslove D. M., Baillie J. K. (2021). Two key takeaways from a year of pandemic research. *Critical Care Medicine*.

[35] Grasselli G., Pesenti A., Cecconi M. (2020). Critical care utilization for the COVID-19 outbreak in Lombardy, Italy. *JAMA*.

[36] Helms J., CRICS TRIGGERSEP Group (Clinical Research in Intensive Care and Sepsis Trial Group for Global Evaluation and Research in Sepsis), Tacquard C. (2020). High risk of thrombosis in patients with severe SARS-CoV-2 infection: a multicenter prospective cohort study. *Intensive Care Medicine*.

[37] Docherty A. B., Harrison E. M., Green C. A. (2020). Features of 20 133 UK patients in hospital with COVID-19 using the ISARIC WHO clinical characterisation protocol: prospective observational cohort study. *BMJ*.

[38] Grasselli G., Zangrillo A., Zanella A. (2020). Baseline characteristics and outcomes of 1591 patients infected with SARS-CoV-2 admitted to ICUs of the Lombardy region, Italy. *JAMA*.

[39] Petrilli C. M., Jones S. A., Yang J. (2020). Factors associated with hospital admission and critical illness among 5279 people with coronavirus disease 2019 in New York City: prospective cohort study. *BMJ*.

[40] Lodigiani C., Iapichino G., Carenzo L. (2020). Venous and arterial thromboembolic complications in COVID-19 patients admitted to an academic hospital in Milan, Italy. *Thrombosis Research*.

[41] Feldstein L. R., Rose E. B., Horwitz S. M. (2020). Multisystem inflammatory syndrome in U.S. children and adolescents. *New England Journal of Medicine*.

[42] Hou J., Yang X., Chen C. (2018). Emerging trends and new developments in information science: a document co-citation analysis (2009–2016). *Scientometrics*.

[43] Trujillo C. M., Long T. M. (2018). Document co-citation analysis to enhance transdisciplinary research. *Science Advances*.

[44] Grasselli G., Cattaneo E., Scaravilli V. (2021). Ventilation of coronavirus disease 2019 patients. *Current Opinion in Critical Care*.

[45] Esteban A., Ferguson N. D., Meade M. O. (2008). Evolution of mechanical ventilation in response to clinical research. *American Journal of Respiratory and Critical Care Medicine*.

[46] Aziz S., Arabi Y. M., Alhazzani W. (2020). Managing ICU surge during the COVID-19 crisis: rapid guidelines. *Intensive Care Medicine*.

[47] Marshall J. C., Murthy S., Diaz J. (2020). A minimal common outcome measure set for COVID-19 clinical research. *The Lancet Infectious Diseases*.

[48] Xie J., Wu W., Li S. (2020). Clinical characteristics and outcomes of critically ill patients with novel coronavirus infectious disease (COVID-19) in China: a retrospective multicenter study. *Intensive Care Medicine*.

[49] Du R.-H., Liu L.-M., Yin W. (2020). Hospitalization and critical care of 109 decedents with COVID-19 pneumonia in Wuhan, China. *Annals of the American Thoracic Society*.

[50] Tang N., Bai H., Chen X., Gong J., Li D., Sun Z. (2020). Anticoagulant treatment is associated with decreased mortality in severe coronavirus disease 2019 patients with coagulopathy. *Journal of Thrombosis and Haemostasis*.

[51] Vankrunkelsven H., Verheyen S., Storms G., De Deyne S. (2018). Predicting lexical norms: a comparison between a word association model and text-based word co-occurrence models. *Journal of Cognition*.

[52] Grames E. M., Stillman A. N., Tingley M. W., Elphick C. S. (2019). An automated approach to identifying search terms for systematic reviews using keyword co-occurrence networks. *Methods in Ecology and Evolution*.

[53] Grasselli G., Greco M., Zanella A. (2020). Risk factors associated with mortality among patients with COVID-19 in intensive care units in Lombardy, Italy. *JAMA Internal Medicine*.

[54] Howell B. A. M. (2021). Battling burnout at the frontlines of health care amid COVID-19. *AACN Advanced Critical Care*.

[55] Pollard T. J., Johnson A. E., Raffa J. D., Celi L. A., Mark R. G., Badawi O. (2018). The eICU collaborative research database, a freely available multi-center database for critical care research. *Scientific Data*.

[56] Sun J., Yuan B.-Z. (2020). Bibliometric mapping of top papers in library and information science based on the essential science indicators database. *Malaysian Journal of Library & Information Science*.

[57] Al Husaeni D. F., Nandiyanto A. B. D. (2021). Bibliometric using Vosviewer with publish or perish (using google scholar data): from step-by-step processing for users to the practical examples in the analysis of digital learning articles in pre and post Covid-19 pandemic. *ASEAN Journal of Science and Engineering*.

[58] Baas J., Schotten M., Plume A., Côté G., Karimi R. (2020). Scopus as a curated, high-quality bibliometric data source for academic research in quantitative science studies. *Quantitative Science Studies*.

